# Clozapine Prevents Poly (I:C) Induced Inflammation by Modulating NLRP3 Pathway in Microglial Cells

**DOI:** 10.3390/cells9030577

**Published:** 2020-02-28

**Authors:** Vijayasree V. Giridharan, Giselli Scaini, Gabriela D. Colpo, Tejaswini Doifode, Omar F. Pinjari, Antônio L. Teixeira, Fabricia Petronilho, Danielle Macêdo, João Quevedo, Tatiana Barichello

**Affiliations:** 1 Translational Psychiatry Program, Faillace Department of Psychiatry and Behavioral Sciences, McGovern Medical School, The University of Texas Health Science Center at Houston (UTHealth), Houston, TX 77054, USA; Vijayasree.V.Giridharan@uth.tmc.edu (V.V.G.); Giselli.Scaini@uth.tmc.edu (G.S.); Gabriela.D.Colpo@uth.tmc.edu (G.D.C.); dr.teju.d@gmail.com (T.D.); Omar.F.Pinjari@uth.tmc.edu (O.F.P.); Antonio.L.Teixeira@uth.tmc.edu (A.L.T.); Joao.L.DeQuevedo@uth.tmc.edu (J.Q.); 2 Laboratory of Neurobiology of Inflammatory and Metabolic Processes, Graduate Program in Health Sciences, Health Sciences Unit, University of South Santa Catarina, Tubarão, SC 88700-000, Brazil; fabricia.petronilho@unisul.br; 3 Neuropsychopharmacology Laboratory, Drug Research, and Development Center, Faculty of Medicine, Universidade Federal do Ceará, Fortaleza, CE, Brazil; National Institute for Translational Medicine (INCT-TM, CNPq), Ribeirão Preto, SP 14000-000, Brazil; Danielle.macedo@ufc.br; 4 Translational Psychiatry Laboratory, Graduate Program in Health Sciences, University of Southern Santa Catarina (UNESC), Criciúma, SC 88800-000, Brazil; 5 Neuroscience Graduate Program, The University of Texas MD Anderson Cancer Center UTHealth Graduate School of Biomedical Sciences, Houston, TX 77030, USA; 6 Center of Excellence on Mood Disorders, Faillace Department of Psychiatry and Behavioral Sciences, McGovern Medical School, The University of Texas Health Science Center at Houston (UTHealth), Houston, TX 77054, USA; 7 Laboratory of Experimental Pathophysiology, Graduate Program in Health Sciences, University of Southern Santa Catarina, Criciúma, SC 88800-000, Brazil

**Keywords:** microglia, poly (I:C), inflammation, NLRP3, cytokines, schizophrenia

## Abstract

Schizophrenia is a complex psychiatric disorder that exhibits an interconnection between the immune system and the brain. Experimental and clinical studies have suggested the presence of neuroinflammation in schizophrenia. In the present study, the effect of antipsychotic drugs, including clozapine, risperidone, and haloperidol (10, 20 and 20 μM, respectively), on the production of IL-1α, IL-1β, IL-2, IL-4, IL-5, IL-6, IL-10, IL-17, IL-18, INF-γ, and TNF-α was investigated in the unstimulated and polyriboinosinic-polyribocytidilic acid [poly (I:C)]-stimulated primary microglial cell cultures. In the unstimulated cultures, clozapine, risperidone, and haloperidol did not influence the cytokine levels. Nevertheless, in cell cultures under strong inflammatory activation by poly (I:C), clozapine reduced the levels of IL-1α, IL-1β, IL-2, and IL-17. Risperidone and haloperidol both reduced the levels of IL-1α, IL-1β, IL-2, and IL-17, and increased the levels of IL-6, IL-10, INF-γ, and TNF-α. Based on the results that were obtained with the antipsychotic drugs and observing that clozapine presented with a more significant anti-inflammatory effect, clozapine was selected for the subsequent experiments. We compared the profile of cytokine suppression obtained with the use of NLRP3 inflammasome inhibitor, CRID3 to that obtained with clozapine, to test our hypothesis that clozapine inhibits the NLRP3 inflammasome. Clozapine and CRID3 both reduced the IL-1α, IL-1β, IL-2, and IL-17 levels. Clozapine reduced the level of poly (I:C)-activated NLRP3 expression by 57%, which was higher than the reduction thay was seen with CRID3 treatment (45%). These results suggest that clozapine might exhibit anti-inflammatory effects by inhibiting NLRP3 inflammasome and this activity is not typical with the use of other antipsychotic drugs under the conditions of strong microglial activation.

## 1. Introduction

Schizophrenia (SCZ) is a progressive neurodevelopmental disorder that leads to severe mental illness and it is a major cause of adult disease burden [1]. Changes in cerebral dopaminergic and glutamatergic transmissions are the well-established neurobiological explanations for SCZ pathophysiology [2]. Despite this, increasing evidence from genetic, transcriptome, *postmortem*, peripheral biomarker, and therapeutic studies have postulated that the dysregulation of the immune system actively contributes to SCZ symptoms and progression [3]. Recent reports have demonstrated an imbalance in the host immune response that is associated with the activation of microglia in the pathophysiology of SCZ [4]. Moreover, imaging and *postmortem* studies have reaffirmed the presence of microglial activation in SCZ patients during the acute psychotic phase [5,6].

Cytokines are one of the critical components that orchestrate the immune system homeostasis [7]. Elevated levels of cytokines have been reported in SCZ *postmortem* brain samples [4]. Conversely, evidence also states that lower cytokine levels were found in the brain samples from SCZ individuals [8]. The longitudinal changes in cytokine levels in the context of treatment with antipsychotic medications could explain their anti-inflammatory mechanisms. Several in vitro and in vivo studies have reported the anti-inflammatory effects of antipsychotics drugs. Treatment with chlorpromazine, haloperidol, and risperidone have been shown to reduce the production of proinflammatory cytokines without influencing the levels of the anti-inflammatory interleukin (IL)-10 in lipopolysaccharide-(LPS)-stimulated rat mixed glial cell cultures [9]. Risperidone elicited its anti-inflammatory effects via the inhibition of the microglial activation by reducing the levels of inducible nitric oxide synthase (iNOS), IL-1β, IL-6, and tumor necrosis factor (TNF)-α in interferon (INF)-γ-activated microglia in vitro [10]. Additionally, TNF-α and IL-6 serum levels were suppressed, whereas the IL-10 level was upregulated by clozapine, olanzapine, and risperidone, but not haloperidol in the LPS-treated mice [11]. These pharmacological reports indicate that these antipsychotic drugs also possess anti-inflammatory effects.

An inflammasome is a crucial mediator of responses to physiological and psychological stressors, and the dysregulation of inflammasomes has been implicated in behavioral changes and psychiatric disorders [12,13,14]. Inflammasome activation causes the maturation of caspase-1 and release of cytokines IL-1β and IL-18, which in turn leads to neuroinflammation and neuroimmune modulation [12]. The therapeutic potential of a NOD-like receptor (NLR) family and pyrin domain-containing protein-3 (NLRP3) inflammasome inhibitor has been demonstrated in autoinflammatory and autoimmune diseases [15]. The results from by Qiao et al., revealed that hepatic NLRP3 inflammasome inhibition reduces the levels of inflammatory cytokines in the brain and thereby delays the progression of dopaminergic neuronal degeneration [16]. A recent study that was conducted on the *postmortem* brain samples from bipolar disorder (BD) and SCZ patients revealed an immune activation in the frontal cortex in both diseases [17]. In BV-2 microgllia that was stimulated with hemozoin, NLRP3 inhibitor (CRID3) was shown to inhibit IL-1β, NO/iNOS, caspase-1, and NLRP3 activity, but not TNF-α and IL-6. Hence, it is essential to understand the neurobiological mechanisms underlying the inhibitory effect of antipsychotics on inflammasome activation and compare it with NLRP3 inhibitor. Thus, this study aimed to investigate the effects of clozapine, risperidone, and haloperidol on the cytokine levels in primary microglial cells activated by polyriboinosinic-polyribocytidilic acid [poly (I:C)], which mimics viral infection. After determining the cytokine levels, we subsequently evaluated a possible association of the NLRP3 pathway with the anti-inflammatory mechanisms of action by comparing the effect of clozapine with a potent NLRP3 inhibitor (CRID3 sodium salt).

## 2. Materials and Methods

### 2.1. Materials

Poly (I:C) was purchased from InvivoGen (San Diego, CA, USA). Clozapine, risperidone, haloperidol, and CRID3 (CP-456, 773) were purchased from Sigma–Aldrich (St. Louis, MO, USA). The antipsychotics risperidone (20 μM), clozapine (10 μM), and haloperidol (20 μM) were dissolved in dimethyl sulfoxide (DMSO) with a final concentration of 0.05% in the culture medium [18,19]. DMSO at the highest concentration (0.05%) that was used for the experimental conditions was not toxic to the cells.

### 2.2. Cell Cultures

#### 2.2.1. Cell Viability Assay

Cell viability was determined by using a 3-(4,5-dimethylthiazol-2-yl)-2,5-diphenyltetrazolium bromide (MTT, Sigma–Aldrich) reduction assay, as previously described with minor modifications [20]. The microglial cells were plated into 96-well plates at a density of 5 × 10^4^ cells/mL, followed by treatment with different concentrations of clozapine, risperidone, haloperidol, and CRID3, as described above. After 24 h of treatment, 0.5 mg/mL MTT was added to each well and then incubated for 2 h at 37 °C. The formazan crystals in the cells were solubilized with 200 μL DMSO. The optical density was quantified at 560–630 nm by a microplate reader (Biotek, Winooski, VT, USA). Cell viability is reported as a percentage ratio of the absorbance of exposed cells to that of the vehicle cells.

#### 2.2.2. Primary Microglial Cultures

**Figure 1:**
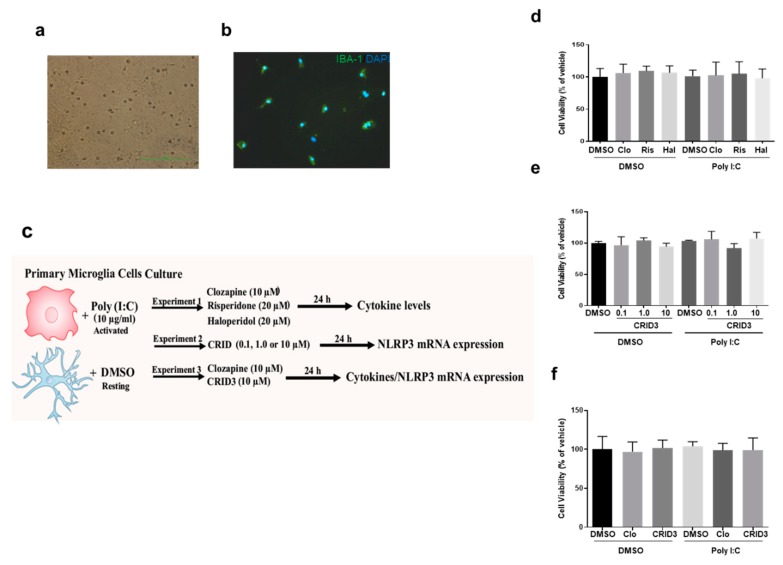
Mixed culture of glial cells from postnatal rat brains showing microglial cells as small round cells on top of the astrocytic monolayer, scale bar = 1 mm (**a**). Cell-type characterization of primary microglial cell cultures. Microglial cells identified using anti-Iba-1 primary and fluorescein isothiocyanate (FITC)-conjugated secondary antibodies (**b**). The schematic representation of the experimental design (**c**). 3-(4,5-dimethylthiazol-2-yl)-2,5-diphenyltetrazolium bromide (MTT) assay with antipsychotics (clozapine (CLO), risperidone (RIS), haloperidol (HAL)) (**d**). MTT assay with CRID3 (0.1, 1.0 or 10 μM) (**e**). MTT assay with 10 μM CLO and 10 μM CRID3 (**f**). Following treatment with different concentrations of CLO, RIS, HAL, or CRID3, MTT (0.25 mg/mL) was added to each well and incubated at 37 °C for 24 h. The experiments were performed with each sample in triplicate. The data is expressed as the mean ± SD of the three independent experiments.

The primary murine microglial cells were collected from one to three days-old Wistar neonatal rats (Both male and female). Pregnant Wistar rats were purchased from Charles River Laboratories. All of the animal manipulations were conducted in accordance with the Guidelines for Animal Experimentation of the The University of Texas Health Science Center at Houston (AWC-15-0056). The neonates were anesthetized by hypothermia (placing on ice) and then decapitated. The meninges were removed under aseptic conditions, and the brain cortices were minced and dissociated with 0.25% trypsin/0.5 mM EDTA. The dissociated cells were then passed through a 70 μM nylon cell strainer (Falcon, USA). The cells were then collected by centrifugation, followed by resuspension in Dulbecco’s modified Eagle’s medium (DMEM) 1× + Glutamax (10566-016, Gibco by Life Technologies, Gaithersburg, MD, USA) containing antibiotics (100 U/mL penicillin and 100 μg/mL streptomycin) (15140-122, Gibco by Life Technologies) and 10% FBS (10082-147, Gibco by Life Technologies, Gaithersburg, MD, USA), and they were cultured in T-75 flasks in 5% CO_2_ at 37 °C. The culture medium was changed twice weekly. After 15 days, the mixed cultures were completely confluent, and the cells were then shaken at 230 rpm for 2–3 h at 37 °C. The detached cells were centrifuged at 2500× *g* for 5 min. The cell pellets were resuspended in microglia medium (1901, ScienCell, Carlsbad, CA, USA) and plated at a density of 2 × 10^5^ cells/cm^2^ (Figure 1a,b).

#### 2.2.3. Cell Treatment

For experiment 1, the microglia cells were exposed to poly (I:C) (10 μg/mL) to stimulate an immune challenge, and immediately after the challenge, the antipsychotic drugs clozapine (10 μM), risperidone (20 μM), or haloperidol (20 μM) were added for 24 h at 37 °C in 5% CO_2_ atmosphere. For experiment 2, the microglia cells were exposed to poly (I:C) (10 μg/mL) and then immediately followed by treatment with CRID3 at concentrations of 0.1, 1.0, or 10 μM for 24 h at 37 °C in a 5% CO_2_ atmosphere. For experiment 3, the microglia cells were exposed to poly (I:C) (10 μg/mL) and immediately followed by treatment with clozapine (10 μM) and CRID3 (10 μM) for 24 h at 37 °C in a 5% CO_2_ atmosphere. The experiments were performed in biological triplicates, and the samples were used as technical duplicates. Figure 1c shows the schematic representation of the experimental design. These final concentrations were chosen based on previous antipsychotic cell culture studies [10,15,21,22].

### 2.3. Multiplex Assay for the Quantification of the Inflammatory Cytokines

The cytokine levels were measured while using multiplex fluorescent immunoassay kits (Bio-Plex Pro™ Rat Cytokine 14-Plex Assay, Mount Joy, PA, USA) [23]. The xMAP platform used here was based on Rules-Based Medicine (RBM) fluorescent beads and antibody pairs. These are sensitive, specific, and widely used reagents that are made by numerous manufacturers. The data that were collected using xMAP multiplex beads were widely reported in the literature, particularly in studies in which multiple proteins are assayed simultaneously. Cell lysates were prepared according to the instructions provided by a Bio-Plex Cell Lysis kit (#171304011) with a protease inhibitor cocktail (Sigma-Aldrich, St. Louis, MO, USA) and then centrifuged at 4 °C for 10 min. at 10,000× *g*. The assays were conducted in 96-well polystyrene, round-bottom microplates, following manufacturer’s instructions. After prior optimization, samples were assessed undiluted in a single-blind experiment, and run in duplicate using a Bioplex system (Bio-Plex 200 Systems, BioRad, Hercules, CA, USA). The data for cytokines were analzed using Bio-Plex Manager™ 4.0 software (Bio-Rad). Among the several models available, we used five-parameter logistic regression model (5PL) with weighting to obtain the standard curve that gives high dynamic range to fit more samples. The calculated values within the range of 70–130% of expected levels were accepted [24].

### 2.4. Gene Expression

Total RNA was isolated from microglia cells using an RNeasy Plus Mini kit (Qiagen, Hilden, Germany) according to the manufacturer’s instructions, and the concentrations were assessed using a NanoDrop (Thermo Scientific Pierce, Waltham, MA, USA). After, the high-Capacity cDNA Reverse Transcription kit (Applied Biosystems, Foster City, CA, USA) was used to converted RNA into complementary DNA (cDNA), according to the manufacturer’s instructions. The reactions were carried out for 10 min. at 25 °C, 2 h at 37 °C, and 5 s at 85 °C. The mRNA levels of genes involved in the inflammasome (NLRP3, ASC, and Casp1) were evaluated by real-time PCR using specific TaqMan FAM/MGB assays (Applied Biosystems, ID assay Rn04244620_m1 for NLRP3, ID assay Rn00597229_g1 for ASC, and ID assay Rn00562724_m1 for Casp1). Rat GAPDH Endogenous Control VIC ⁄ MGB (Applied Biosystems, Beverly, MA, USA; 4352338E) were used to normalized the transcript levels. The reactions were performed in an Applied Biosystems 7500 Real-Time PCR System, which detects the PCR product directly without downstream processing. The reactions were carried out in a total volume of 12 µL with 6 µL 2× TaqMan Gene Expression Master Mix (containing ROX, Amplitaq Gold DNA polymerase, AmpErase UNG, dATP, dCTP, dGTP, dUTP and MgCl2), 0.6 µL 20× TaqMan Gene Expression Assay, 0.6 µL 20× TaqMan Endogenous Control, 3.8 µL water, and 1 µL cDNA solution. The cycling program consisted of 2 min. at 50 °C and 10 min. at 95 °C followed by 40 cycles of 15 s at 95 °C and 1 min. at 60 °C. All reactions were performed in triplicate. The relative expression levels were determined by the ddCt method, as described by Livak and Schmittgen (2001) [25].

### 2.5. Caspase-1 Activity Assay

The assay was carried out while using the Caspase-1/ICE colorimetric assay kit (BioVision, Milpitas, CA, USA) in 96-well plates. The cell lysates were prepared using cell lysis buffer provided by the kit. Cell lysates (50–200 μg) were then incubated with 4 mM YVAD-pNA substrate (200 µM final concentration), according to the manufacturer’s instruction. After 1 h of incubation at 37 °C, the absorbance was read on BioTek’s Synergy™ H1 Multi-Mode Microplate Reader at 405 nm. Caspase activity in microglia cells that were treated with DTT 10 mM was used as a positive control.

### 2.6. Statistical Analysis

The results are presented as the mean ± standard deviation (SD). Comparisons between multiple groups were made while using one-way ANOVA followed by Tukey’s *post hoc* analysis. The experiments were performed with each sample in triplicate. Significance was set at *p* < 0.05. All of the statistical analyses were performed while using GraphPad Prism 7.0 software (GraphPad Software, Inc., La Jolla, CA, USA).

## 3. Results 

### 3.1. Cell Viability Assay

The MTT assay was performed to evaluate the cytotoxicity of clozapine, risperidone, haloperidol, and CRID3 in microglial cell culture. At the concentrations used, co-treatment with clozapine (10 μM), risperidone (20 μM), haloperidol (20 μM), and CRID3 (0.1, 1.0 and 10.0 μM) did not affect the cell viability, as shown in Figure 1d–f.

### 3.2. The Effect of Antipsychotic Drugs on Poly (I:C)-Induced Cytokine Levels in Primary Microglial Cell Cultures

**Figure 2:**
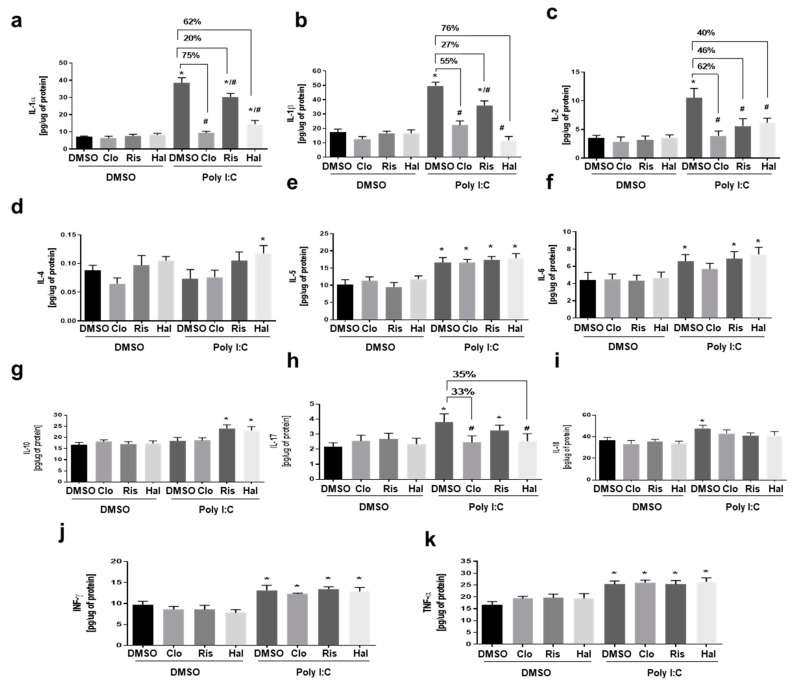
The effect of antipsychotics on poly (I:C)-induced cytokine levels: The effect of antipsychotics on (**a**) IL-1α, (**b**) IL-1β, (**c**) IL-2, (**d**). IL-4, (**e**). IL-5, f. IL-6, (**g**) IL-10, (**h**) IL-17, (**i**) IL-18, (**j**) INF-γ, and (**k**). TNF-α cytokines in poly (I:C)-stimulated microglia. Microglia were exposed to poly (I:C) (10 μg/mL) to simulate viral stimulation and then immediately treated with antipsychotics for 24 h. The experiments were performed with each sample in triplicate. clozapine (CLO), risperidone (RIS), haloperidol (HAL). The data are expressed as the mean ± SD of three independent experiments. * *p* < 0.05 vs. control samples. # *p* < 0.05 vs. poly (I:C)-treated samples.

We evaluated the anti-inflammatory effect of the antipsychotic drugs by determining the cytokine levels (IL-1α, IL-1β, IL-2, IL-4, IL-5, IL-6, IL-10, IL-17, IL-18, INF-γ, and TNF-α) in primary microglial cell cultures activated by poly (I:C). Figure 2 shows that the levels of IL-1α, IL-1β, IL-2, IL-5, IL-6, IL-17, IL-18, INF-γ, and TNF-α in the poly (I:C)-treated cultures were upregulated when compared to the cytokine levels in the medium harvested from the cultures that were treated with vehicle (0.05% DMSO). Clozapine at a 10 µM concentration reduced poly (I:C)-induced IL-1α, IL-1β, IL-2, and IL-17 levels by 75%, 55%, 62% and 33%, respectively. However, clozapine did not affect the IL-6 and IL-10 levels. Risperidone at a 20 µM concentration reduced the poly (I:C)-induced IL-1α, IL-1β, IL-2, and IL-17 levels by 20%, 27%, and 46%, respectively. Risperidone also increased the levels of IL-6, IL-10, INF-γ, and TNF-α when compared to the vehicle. Haloperidol at a 20 µM concentration reduced the poly (I:C)-induced IL-1α, IL-1β, IL-2, and IL-17 levels by 62%, 76%, 40%, and 35%, respectively. Similar to risperidone, haloperidol also increased the levels of IL-6, IL-10, INF-γ, and TNF-α when compared to the vehicle, as demonstrated in Figure 2. Based on the results that were obtained with the antipsychotic drugs and observing that clozapine presented a more significant inhibitory action against poly (I:C)-induced IL-1α, IL-1β, IL-2, and IL-17 production, clozapine was selected for the subsequent experiments.

### 3.3. The Effect of NLRP3 Inflammasome Inhibitor on Poly (I:C)-Induced Cytokine Levels in Primary Microglial Cell Cultures

**Figure 3:**
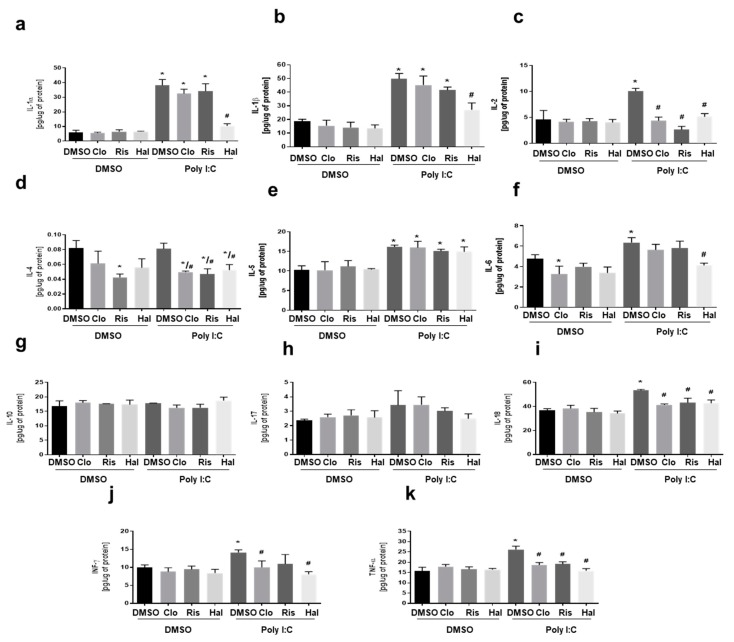
The effect of CRID3 on poly(I:C)-induced cytokine levels: The effect of CRID3 on the expression of (**a**) IL-1α, (**b**) IL-1β, (**c**) IL-2, (**d**) IL-4, (**e**) IL-5, (**f**) IL-6, (**g**) IL-10, (**h**) IL-17, (**i**) IL-18, (**j**) INF-γ, and (**k**) TNF-α cytokines in poly (I:C)-stimulated microglia. The microglia were exposed to poly (I:C) (10 μg/mL) and then immediately treated with CRID3 at concentrations of 0.1, 1.0, or 10 μM for 24 h. Experiments were performed with each sample in triplicate. clozapine (CLO), risperidone (RIS), and haloperidol (HAL). The data is expressed as the mean ± SD of the three independent experiments. * *p* < 0.05 vs. control samples. # *p* < 0.05 vs. poly (I:C)-treated samples.

In the next study, we evaluated the most effective concentration of CRID3, a specific NLRP3 inflammasome inhibitor, in microglial cell cultures that were activated by poly (I:C). Although in vitro [26] and in vivo [27] studies have demonstrated the effect of CRID3 on inflammasome activation, to the best of our knowledge, this is the first attempt to investigate the effect of CRID3 on poly (I:C)-induced cytokine production in primary microglial cells culture. In experiment 2, CRID3 at a higher concentration (10 μM) significantly reduced poly (I:C)-induced IL-1α, IL-1β, IL-2, IL-4, IL-6, IL-18, INF-γ, and TNF-α levels, as demonstrated in Figure 3.

### 3.4. The Effects of Clozapine and CRID3 on Poly (I:C)-Induced Cytokine Levels in Primary Microglial Cell Cultures

**Figure 4:**
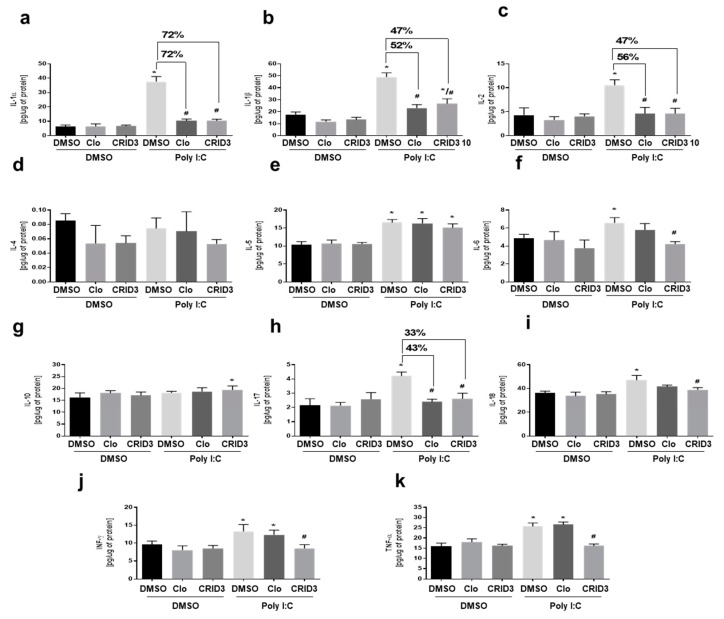
The effect of clozapine and CRID3 on poly (I:C) induced cytokine levels: The effects of 10 μM clozapine and 10 μM CRID3 on the expression of (**a**) IL-1α, (**b**) IL-1β, (**c**) IL-2, (**d**) IL-4, (**e**) IL-5, (**f**) IL-6, (**g**) IL-10, (**h**) IL-17, (**i**) IL-18, (**j**) INF-γ, and (**k**) TNF-α cytokines in poly (I:C)-stimulated microglia. The microglia were exposed to poly (I:C) (10 μg/mL) and then immediately treated with clozapine (10 μM) and CRID3 (10 μM) for 24 h. The experiments were performed with each sample in triplicate. clozapine (CLO). The data is expressed as the mean ± SD of three independent experiments. *p < 0.05 vs. control samples. # *p* < 0.05 vs. poly (I:C)-treated samples.

Next, experiment 3 was performed to compare the effects of clozapine (10 μM) and CRID3 (10 μM) on poly (I:C)-induced cytokine levels in primary microglia cells culture. Clozapine and CRID3 both reduced poly (I:C)-induced IL-1α, IL-1β, IL-2, and IL-17 levels, as demonstrated in Figure 4. Poly (I:C)-induced IL-1α was reduced to 72% by both clozapine and CRID3. We found reduction in the levels of IL-1β to 52% and 47% by clozapine and CRID3, respectively. The levels of IL-2 were reduced to 56% by clozapine as compared to and CRID3 (47%). We also found the reduced levels of IL-17 by clozapine and CRID3 to 43% and 33%, respectively. The poly (I:C)-induced increases in IL-6, IL-18, INF-γ, and TNF-α levels were significantly reduced by CRID3.

### 3.5. The Effect of Clozapine and CRID3 on Poly (I:C)-Induced NLRP-3 Inflammasome Activation in Primary Microglia

**Figure 5:**
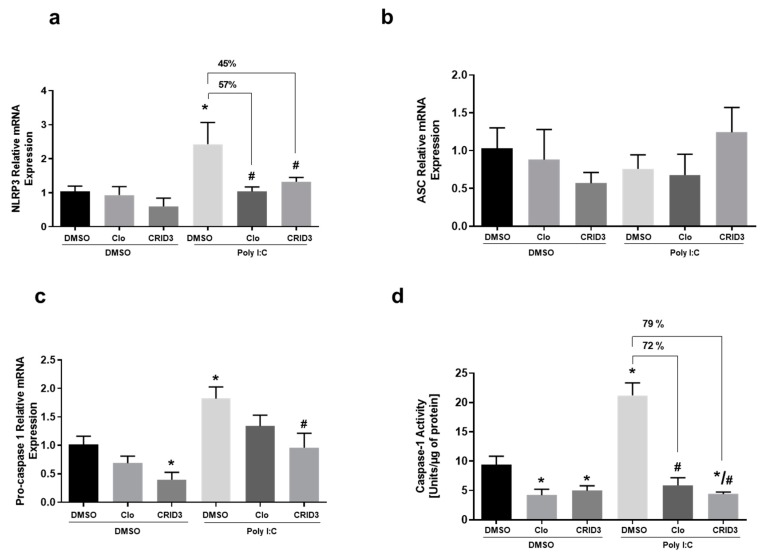
The effect of clozapine and CRID3 on poly (I:C)-induced inflammasomes: The effects of 10 μM clozapine and 10 μM CRID3 on the mRNA expression of (**a**) NLRP3, (**b**) ASC, (**c**) procaspase-1, and (**d**) Caspase-1 activity. The microglia were exposed to poly (I:C) (10 μg/mL) and then immediately treated with clozapine (10 μM) and CRID3 (10 μM) for 24 h. The experiments were performed with each sample in triplicate. clozapine (CLO). The data is expressed as the mean ± SD of three independent experiments. * *p* < 0.05 vs. control samples. # *p* < 0.05 vs. poly (I:C)-treated samples.

Furthermore, we investigated the mRNA expression of the NLRP3-related proteins NLRP3, ASC, pro-caspase 1, and caspase-1 activity, after clozapine or CRID3 treatment in primary microglial cell cultures, to evaluate whether the anti-inflammatory effects of clozapine involve the NLRP3 pathway. As expected, poly (I:C) significantly elevated the NLRP3, pro-caspase-1 levels, as demonstrated in Figure 5. Interestingly, the antipsychotic drug clozapine significantly reduced the levels of poly (I:C)-activated NLRP3 expression by 57%, which was higher than the level of NLRP3 inhibitor, CRID3 (45%). We found no difference in the mRNA expression levels of ASC. However, the poly (I:C)-induced pro-caspase 1 levels were only significantly reduced by CRID3. The poly (I:C)-induced caspase 1 activity was significantly reduced by both clozapine (72%) and CRID3 (79%).

## 4. Discussion

This study demonstrates that the antipsychotic drugs clozapine, risperidone, and haloperidol influence the balance between pro- and anti-inflammatory cytokines upon cell stimulation by poly (I:C). Among the tested antipsychotic drugs, clozapine profoundly reduced the expression of the proinflammatory cytokines and significantly inhibited NLRP3 inflammasome activation, and its effect was comparable to CRID3, which is an NLRP3 inflammasome inhibitor. 

Prenatal or neonatal challenges with poly (I:C), a TLR-3 agonist, have been widely accepted as a neurodevelopmental animal model of SCZ [28,29]. Recently, this approach was also used to generate a standard model of viral infection to induce microglial activation [30,31]. The Poly (I:C) model successfully accounts for several aspects of SCZ, including epidemiology, pathophysiology, symptomatology, and treatment. Several studies have supported the poly (I:C) experimental model as a very powerful neurodevelopmental animal model of SCZ and relevant brain disease. Additionally, the poly (I:C) model that is used for exploring of novel pharmacological targets presumably considered for SCZ and related disorders and this is the primary reason to use poly (I:C) model in this study [32]. We used poly (I:C) to stimulate the rat primary mixed glial cell cultures enriched for microglia to mimic the SCZ condition in vitro. We demonstrated that the tested antipsychotics at the given doses showed anti-inflammatory effects with no cell toxicity. The concentrations for all antipsychotic drugs that were used in conjunction with poly (I:C) stimulation were identified by a review of the published literature which used in vitro models [18,19]. 

Among clozapine, risperidone, and haloperidol, the three tested antipsychotics, clozapine showed profound proinflammatory inhibitory action by reducing the levels of IL-1α, IL-1β, IL-2, and IL-17. Clinically in the blood and CSF of SCZ patients, alterations in cytokine concentrations, cytokine receptors, and their activity have been reported [33,34] Our results were consistent with an earlier report showing a proinflammatory inhibitory profile of clozapine in an animal model [11]. 

To date, the atypical antipsychotic drug clozapine, 3-chloro-6-(4-methylpiperazin-1-yl)-5H-benzo[b][1,4]benzodiazepine, is widely accepted as a ‘gold standard’ for the treatment of SCZ [35,36,37,38]. Specifically, clozapine is the most effective drug for treatment-resistant SCZ patients, with the potential added benefits of reducing suicide risk and aggression. Clozapine exerts its anti-inflammatory effect by modulating the cytokine levels that were stimulated by LPS and poly (I:C) in mice model [11] and microglial cells [39] and also in poly (I:C) stimulated peripheral blood mononuclear cells (PBMC) from SCZ patients [40]. There is also some evidence that clozapine exhibits anti-inflammatory activity through reducing toll-like receptor (TLR)-4/ nuclear factor (NF)-κB-mediated inflammatory responses through the inhibition of calcium/calmodulin-dependent Akt activation [39]. Clozapine protects dopaminergic neurons from inflammation-induced damage via the inhibition of microglial overactivation through the phosphoinositide 3-kinase (PI3K) pathway [18]. However, the effect of clozapine on inflammasome inhibition has not been demonstrated. 

The antagonism of D2 and serotonin type 2A (5-HT2A) receptors mediate the therapeutic effect of clozapine in SCZ [41,42]. Recent evidence from PBMC cultures in SCZ patients revealed the anti-inflammatory effect of clozapine on LPS- and poly (I:C)-induced inflammatory responses [40]. Although the mechanism of action of clozapine has been explained by the dopaminergic theory and the immune regulatory response that suppresses inflammation, the exact mechanism of action of clozapine has not yet been fully elucidated.

The prototypical inflammasome is formed by the Nod-like receptor protein, NLRP3 interacting with an adapter molecule, apoptosis-associated speck-like protein containing a CARD (ASC) via the Pyrin domain (PYD) of NLRP3. The caspase activation and recruitment domain (CARD) of ASC, in turn, binds with the CARD domain of caspase-1 [43]. Inflammasomes are assembled in response to a number of exogenous and endogenous danger signals, which in turn, leads to the production of proinflammatory cytokines. Ultimately, through the activation of caspase-1, inflammasome activation leads to the induction of inflammatory cell death. Thus, inflammasomes have been implicated in a wide range of physiological and pathological processes that can be both beneficial and detrimental. Hence, understanding the mechanisms that are involved in inflammasome activation might provide a better approach in preventing the harmful effects of the inflammatory response [44]. In the last few decades, the importance of NLRP3 inflammasome biology has become more apparent in inflammatory diseases, such as Alzheimer’s disease [45], stroke [46], inflammatory bowel diseases [47], and atherosclerosis [48]. It is interesting to note that these deleterious conditions were improved by the NLRP3 inhibitor CRID3 [45,49]. Additionally, NLRP3 plays a crucial role in immune sensing within the innate immune system. The activation of the NLRP3 inflammasome is also implicated in the pathogenesis of chronic diseases and aging. In psychiatric disorders, postmortem brain samples from bipolar patients demonstrated the activation of NLRP3 as compared to controls [17].

CRID3, a novel inflammasome inhibitor, exerts its action by inhibiting the NLRP3 and absent in melanoma (AIM) 2 inflammasomes by preventing ASC oligomerization. CRID3 specifically targets glutathione S-transferase omega 1 (GSO1) and regulates caspase-1 activation and the production of the proinflammatory cytokine IL-1β [26]. In this study, we found that treatment with the inflammasome inhibitor CRID3 at a 10 µM concentration resulted in the inhibition of the expression of the proinflammatory cytokines IL-1α, IL-1β IL-2, IL-4, IL-6, IL-18, INF-γ, and TNF-α in poly (I:C)-stimulated microglia. The results demonstrated that both clozapine and CRID3 exert anti-inflammatory effects by reducing the proinflammatory cytokine levels in poly (I:C)-stimulated microglia. We further examined the protective effect of clozapine on NLRP3 inflammasome activation by measuring the mRNA levels. It was interesting to note that clozapine reduced the level of NLRP3 expression by 57%, which was higher than the reduction that was seen with the NLRP3 inflammasome inhibitor CRID3 (45%).

## 5. Conclusions

In conclusion, our study demonstrated that clozapine suppresses the proinflammatory cytokine expression by limiting the NLRP3 inflammasome activation in an in vitro model. Further in vivo studies are necessary to open a new avenue for the potential pharmacological use of clozapine in NLRP3 inflammasome-driven inflammatory diseases.

## References

[1] Charlson F.J., Ferrari A.J., Santomauro D.F., Diminic S., Stockings E., Scott J.G., McGrath J.J., Whiteford H.A. (2018). Global Epidemiology and Burden of Schizophrenia: Findings From the Global Burden of Disease Study 2016. Schizophr. Bull..

[2] Howes O., McCutcheon R., Stone J. (2015). Glutamate and dopamine in schizophrenia: An update for the 21st century. J. Psychopharmacol..

[3] Birnbaum R., Jaffe A.E., Chen Q., Shin J.H., Kleinman J.E., Hyde T.M., Weinberger D.R. (2018). Investigating the neuroimmunogenic architecture of schizophrenia. Mol. Psychiatry.

[4] Horvath S., Mirnics K. (2014). Immune system disturbances in schizophrenia. Biol. Psychiatry.

[5] Selvaraj S., Bloomfield P.S., Cao B., Veronese M., Turkheimer F., Howes O.D. (2018). Brain TSPO imaging and gray matter volume in schizophrenia patients and in people at ultra high risk of psychosis: An [(11)C]PBR28 study. Schizophr. Res..

[6] Kenk M., Selvanathan T., Rao N., Suridjan I., Rusjan P., Remington G., Meyer J.H., Wilson A.A., Houle S., Mizrahi R. (2015). Imaging neuroinflammation in gray and white matter in schizophrenia: An in-vivo PET study with [18F]-FEPPA. Schizophr. Bull..

[7] Girgis R.R., Kumar S.S., Brown A.S. (2014). The cytokine model of schizophrenia: Emerging therapeutic strategies. Biolo. Psychiatry.

[8] Trepanier M.O., Hopperton K.E., Mizrahi R., Mechawar N., Bazinet R.P. (2016). Postmortem evidence of cerebral inflammation in schizophrenia: A systematic review. Mol. Psychiatry.

[9] Obuchowicz E., Bielecka-Wajdman A.M., Paul-Samojedny M., Nowacka M. (2017). Different influence of antipsychotics on the balance between pro- and anti-inflammatory cytokines depends on glia activation: An in vitro study. Cytokine.

[10] Kato T., Monji A., Hashioka S., Kanba S. (2007). Risperidone significantly inhibits interferon-gamma-induced microglial activation in vitro. Schizophr. Res..

[11] Sugino H., Futamura T., Mitsumoto Y., Maeda K., Marunaka Y. (2009). Atypical antipsychotics suppress production of proinflammatory cytokines and up-regulate interleukin-10 in lipopolysaccharide-treated mice. Prog. Neuropsychopharmacol. Biol. Psychiatry.

[12] Wong M.L., Inserra A., Lewis M.D., Mastronardi C.A., Leong L., Choo J., Kentish S., Xie P., Morrison M., Wesselingh S.L. (2016). Inflammasome signaling affects anxiety- and depressive-like behavior and gut microbiome composition. Mol. Psychiatry.

[13] Saresella M., Piancone F., Marventano I., Zoppis M., Hernis A., Zanette M., Trabattoni D., Chiappedi M., Ghezzo A., Canevini M.P. (2016). Multiple inflammasome complexes are activated in autistic spectrum disorders. Brain Behav. Immun..

[14] Kim H.K., Chen W., Andreazza A.C. (2015). The Potential Role of the NLRP3 Inflammasome as a Link between Mitochondrial Complex I Dysfunction and Inflammation in Bipolar Disorder. Neural Plast..

[15] Coll R.C., Robertson A.A., Chae J.J., Higgins S.C., Munoz-Planillo R., Inserra M.C., Vetter I., Dungan L.S., Monks B.G., Stutz A. (2015). A small-molecule inhibitor of the NLRP3 inflammasome for the treatment of inflammatory diseases. Nat. Med..

[16] Qiao C., Zhang Q., Jiang Q., Zhang T., Chen M., Fan Y., Ding J., Lu M., Hu G. (2018). Inhibition of the hepatic Nlrp3 protects dopaminergic neurons via attenuating systemic inflammation in a MPTP/p mouse model of Parkinson’s disease. J. Neuroinflamm..

[17] Kim H.K., Andreazza A.C., Elmi N., Chen W., Young L.T. (2016). Nod-like receptor pyrin containing 3 (NLRP3) in the post-mortem frontal cortex from patients with bipolar disorder: A potential mediator between mitochondria and immune-activation. J. Psychiatr. Res..

[18] Hu X., Zhou H., Zhang D., Yang S., Qian L., Wu H.M., Chen P.S., Wilson B., Gao H.M., Lu R.B. (2012). Clozapine protects dopaminergic neurons from inflammation-induced damage by inhibiting microglial overactivation. J. Neuroimmune Pharmacol..

[19] Souza B.R., Torres K.C., Miranda D.M., Motta B.S., Scotti-Muzzi E., Guimarães M.M., Daniel D.S.C., Daniela V.F.R., Renan P.S., Helton J.R. (2010). Lack of effects of typical and atypical antipsychotics in DARPP-32 and NCS-1 levels in PC12 cells overexpressing NCS-1. J. Negat. Results BioMed..

[20] Mosmann T. (1983). Rapid colorimetric assay for cellular growth and survival: Application to proliferation and cytotoxicity assays. J. Immunol. Methods.

[21] de Oliveira A.C., Yousif N.M., Bhatia H.S., Hermanek J., Huell M., Fiebich B.L. (2016). Poly(I:C) increases the expression of mPGES-1 and COX-2 in rat primary microglia. J. Neuroinflamm..

[22] Kang U.G., Seo M.S., Roh M.S., Kim Y., Yoon S.C., Kim Y.S. (2004). The effects of clozapine on the GSK-3-mediated signaling pathway. FEBS Lett..

[23] Hulse R.E., Kunkler P.E., Fedynyshyn J.P., Kraig R.P. (2004). Optimization of multiplexed bead-based cytokine immunoassays for rat serum and brain tissue. J. Neurosci. Methods.

[24] Baud M., Masseyeff R. (1993). Data Analysis, Mathematical Modeling.

[25] Livak K.J., Schmittgen T.D. (2001). Analysis of relative gene expression data using real-time quantitative PCR and the 2(-Delta Delta C(T)) Method. Methods (San Diego, Calif.).

[26] Coll R.C., Robertson A., Butler M., Cooper M., O’Neill L.A. (2011). The cytokine release inhibitory drug CRID3 targets ASC oligomerisation in the NLRP3 and AIM2 inflammasomes. PLoS ONE.

[27] Ludwig-Portugall I., Bartok E., Dhana E., Evers B.D., Primiano M.J., Hall J.P., Franklin B.S., Knolle P.A., Hornung V., Hartmann G. (2016). An NLRP3-specific inflammasome inhibitor attenuates crystal-induced kidney fibrosis in mice. Kidney Int..

[28] Estes M.L., McAllister A.K. (2016). Maternal immune activation: Implications for neuropsychiatric disorders. Science.

[29] Han M., Zhang J.C., Yao W., Yang C., Ishima T., Ren Q., Ma M., Dong C., Huang X.F., Hashimoto K. (2016). Intake of 7,8-Dihydroxyflavone During Juvenile and Adolescent Stages Prevents Onset of Psychosis in Adult Offspring After Maternal Immune Activation. Sci. Rep..

[30] Suh H.S., Zhao M.L., Derico L., Choi N., Lee S.C. (2013). Insulin-like growth factor 1 and 2 (IGF1, IGF2) expression in human microglia: Differential regulation by inflammatory mediators. J. Neuroinflamm..

[31] Sato-Kasai M., Kato T.A., Ohgidani M., Mizoguchi Y., Sagata N., Inamine S., Horikawa H., Hayakawa K., Shimokawa N., Kyuragi S. (2016). Aripiprazole inhibits polyI:C-induced microglial activation possibly via TRPM7. Schizophr. Res..

[32] Meyer U., Feldon J. (2012). To poly(I:C) or not to poly(I:C): Advancing preclinical schizophrenia research through the use of prenatal immune activation models. Neuropharmacology.

[33] Miller B.J., Buckley P., Seabolt W., Mellor A., Kirkpatrick B. (2011). Meta-analysis of cytokine alterations in schizophrenia: Clinical status and antipsychotic effects. Biol. Psychiatry.

[34] Barak V., Barak Y., Levine J., Nisman B., Roisman I. (1995). Changes in interleukin-1 beta and soluble interleukin-2 receptor levels in CSF and serum of schizophrenic patients. J. Basic Clin. Physiol. Pharmacol..

[35] Taylor D.M. (2017). Clozapine for Treatment-Resistant Schizophrenia: Still the Gold Standard?. CNS Drugs.

[36] Meltzer H.Y. (2013). Update on typical and atypical antipsychotic drugs. Annu. Rev. Med..

[37] Fakra E., Azorin J.M. (2012). Clozapine for the treatment of schizophrenia. Expert Opin. Pharmacother..

[38] Warnez S., Alessi-Severini S. (2014). Clozapine: A review of clinical practice guidelines and prescribing trends. BMC Psychiatry.

[39] Jeon S., Kim S.H., Shin S.Y., Lee Y.H. (2018). Clozapine reduces Toll-like receptor 4/NF-kappaB-mediated inflammatory responses through inhibition of calcium/calmodulin-dependent Akt activation in microglia. Prog. Neuropsychopharmacol. Biol. Psychiatry.

[40] Al-Amin M., Uddin M.M.N., Reza H.M. (2013). Effects of Antipsychotics on the Inflammatory Response System of Patients with Schizophrenia in Peripheral Blood Mononuclear Cell Cultures. Clin. Psychopharmacol. Neurosci..

[41] Schmid C.L., Streicher J., Meltzer H.Y., Bohn L.M. (2014). Clozapine Acts as an Agonist at Serotonin 2A Receptors to Counter MK-801-Induced Behaviors through a βArrestin2-Independent Activation of Akt. Neuropsychopharmacology.

[42] Dziedzicka-Wasylewska M., Faron-Gorecka A., Gorecki A., Kusemider M. (2008). Mechanism of action of clozapine in the context of dopamine D1-D2 receptor hetero-dimerization--a working hypothesis. Pharmacol. Rep. PR.

[43] Narayanan K.B., Jang T.H., Park H.H. (2014). Self-oligomerization of ASC PYD domain prevents the assembly of inflammasome in vitro. Appl. Biochem. Biotech..

[44] Tsuchiya K., Hara H. (2014). The inflammasome and its regulation. Crit. Rev. Immunol..

[45] Dempsey C., Rubio Araiz A., Bryson K.J., Finucane O., Larkin C., Mills E.L., Robertson A.A.B., Cooper M.A., O’Neill L.A.J., Lynch M.A. (2017). Inhibiting the NLRP3 inflammasome with MCC950 promotes non-phlogistic clearance of amyloid-beta and cognitive function in APP/PS1 mice. Brain Behav. Immun..

[46] Ren H., Kong Y., Liu Z., Zang D., Yang X., Wood K., Li M., Liu Q. (2018). Selective NLRP3 (Pyrin Domain-Containing Protein 3) Inflammasome Inhibitor Reduces Brain Injury After Intracerebral Hemorrhage. Stroke.

[47] Neudecker V., Haneklaus M., Jensen O., Khailova L., Masterson J.C., Tye H., Biette K., Jedlicka P., Brodsky K.S., Gerich M.E. (2017). Myeloid-derived miR-223 regulates intestinal inflammation via repression of the NLRP3 inflammasome. J. Exp. Med..

[48] van der Heijden T., Kritikou E., Venema W., van Duijn J., van Santbrink P.J., Slutter B., Foks A.C., Bot I., Kuiper J. (2017). NLRP3 Inflammasome Inhibition by MCC950 Reduces Atherosclerotic Lesion Development in Apolipoprotein E-Deficient Mice-Brief Report. Arter. Thromb. Vasc. Biol..

[49] Mangan M.S., Olhava E.J., Roush W.R., Seidel H.M., Glick G.D., Latz E. (2018). Targeting the NLRP3 inflammasome in inflammatory diseases. Nat. Rev..

